# The global regulators ArcA and CytR collaboratively modulate *Vibrio cholerae* motility

**DOI:** 10.1186/s12866-022-02435-y

**Published:** 2022-01-12

**Authors:** Yuehua Li, Junxiang Yan, Xueqian Guo, Xiaochen Wang, Fenxia Liu, Boyang Cao

**Affiliations:** 1 TEDA Institute of Biological Sciences and Biotechnology, Nankai University, No.23, Hongda Street, Tianjin Economic and Technological Development Area, Tianjin, 300457 China; 2 Key Laboratory of Molecular Microbiology and Technology of the Ministry of Education, Nankai University, No.23, Hongda Street Tianjin Economic and Technological Development Area, Tianjin, 300457 China; 3 Tianjin Key Laboratory of Microbial Functional Genomics, TEDA College, Nankai University, No.23, Hongda Street Tianjin Economic and Technological Development Area, Tianjin, 300457 China; 4 TEDA Institute of Biological Sciences and Biotechnology, Nankai University, No.23, Hongda Street Tianjin Economic and Technological Development Area, Tianjin, 300457 China; 5 Key Laboratory of Molecular Microbiology and Technology of the Ministry of Education, Nankai University, No.23, Hongda Street Tianjin Economic and Technological Development Area, Tianjin, 300457 China; 6 Tianjin Key Laboratory of Microbial Functional Genomics, TEDA College, Nankai University, No.23, Hongda Street Tianjin Economic and Technological Development Area, Tianjin, 300,457 China

**Keywords:** Vibrio cholerae, Motility, ArcA, CytR, Flagellum

## Abstract

**Background:**

Vibrio cholerae, a Gram-negative bacterium, is highly motile owing to the presence of a single polar flagellum. The global anaerobiosis response regulator, ArcA regulates the expression of virulence factors and enhance biofilm formation in V. cholerae. However, the function of ArcA for the motility of V. cholerae is yet to be elucidated. CytR, which represses nucleoside uptake and catabolism, is known to play a chief role in V. cholerae pathogenesis and flagellar synthesis but the mechanism that CytR influences motility is unclear.

**Results:**

In this study, we found that the ΔarcA mutant strain exhibited higher motility than the WT strain due to ArcA directly repressed flrA expression. We further discovered that CytR directly enhanced fliK expression, which explained why the ΔcytR mutant strain was retarded in motility. On the other hand, cytR was a direct ArcA target and cytR expression was directly repressed by ArcA. As expected, cytR expression was down-regulated.

**Conclusions:**

Overall, ArcA plays a critical role in V. cholerae motility by regulating flrA expression directly and fliK indirectly in the manner of cytR.

**Supplementary Information:**

The online version contains supplementary material available at 10.1186/s12866-022-02435-y.

## Background

V. cholerae is ubiquitous in aquatic environments and intestines of host [1]. The flagellum of V. cholerae is a complex self-assembling organelle that is attached to the cell surface and allows bacterial cells to move in their environment [2]; moreover, it plays a pivotal role in substrates adhesion, biofilm formation, and virulence [3–6]. Elucidating the mechanisms underlying the regulation of flagellum should enhance our understanding of the lifecycle of *V. cholerae* both in the intestinal and aquatic phases. *V. cholerae* flagellar genes are expressed within a fourtiered transcriptional hierarchy [4]. The sole Class I gene encodes the σ54-dependent transcriptional activator FlrA, which is the master regulator of the flagellar hierarchy because without it no flagellar genes are expressed [7]. FliK is in the Class III level of the flagellar hierarchy. The assembly of a flagellum occurs in a number of stages, and FliK is the ‘‘checkpoint control’’ protein When the flagellar hook substructure has reached its optimal length, FliK then terminates hook export and assembly and transmits a signal to begin filament export, in the final stage of the flagellar biosynthesis [8].

The ArcA/ArcB two-component system evidently has a key role in the response to oxygen. The membrane sensor protein ArcB is phosphorylated, resulting in the transfer of its phosphoryl group to ArcA. Phosphorylated ArcA is subsequently activated as a transcription factor, resulting in the up-or downregulation of several downstream genes [9, 10]. In *Escherichia coli*, approximately 50% genes whose expression levels are affected due to aerobic to anaerobic transitions are also affected by ArcA; in total 1139 genes in the *E. coli* genome are in fact either directly or indirectly regulated by ArcA [11]. In *Salmonella* Typhimurium, ArcA has been shown to affect metabolic processes, stress response, and surface adherence [12]. A study reported that the Δ*arcA* mutant strain lacked flagella in *S. enterica sv.* Typhimurium and was thus non-motile [13]. In *E. coli*, ArcA enhances motility by increasing *fliA* expression [14]. Further ArcA was found to directly repress the expression of *motA*, *motB*, and *cheA* in avian pathogenic *E. coli*, but it did not affect the expression of flagella genes [15]. Considering that the function of ArcA in *V. cholerae* motility remains unexplored, in this study, we aimed to determine the relationship between ArcA and *V. cholerae* motility.

The CytR repressor belongs to the LacI family, and possesses, like the other members, an N-terminal helix–turn–helix (HTH) DNA-binding motif [16]. However, unlike a typical bacterial repressor, the CytR repressor and the cAMP receptor protein (CRP) bind cooperatively to several promoters in *E. coli* to repress transcription initiation [17]. In addition, researches had shown that the CRP protein and the CytR regulator can act either to repress or to activate transcription depending on the context [18]. CytR negatively regulates the genes that are involved in nucleosides uptake and catabolism [19]. In uropathogenic *E. coli*, CytR is a modulator of flagellar expression activated by CRP. The Δ*cytR* mutant strain was observed to show higher motility and flagellar expression [20]; further, CytR bound to the upstream region of *flhD*, which encodes the master regulator for flagellar expression. In *V. cholerae*, CytR positively regulates competence genes, type VI secretion operons, and chitinases [21]. Moreover, it plays an important role in *V. cholerae* pathogenesis and flagella synthesis [22]. In *V. cholrae,* the Δ*cytR* mutant strain was found to show downregulated expression levels of the class II flagellar genes *flrB* and *flrC*, and several class III flagellar genes [22]. Nevertheless, further studies are warranted to comprehensively understand this process. In the current study, we identified that low oxygen levels enhanced *arcA* expression, but repressed *cytR* expression. Furthermore, ArcA reduces *V. cholerae* motility not only in the manner of directly regulating *flrA*, but also in the manner of indirectly regulating *fliK* via directly binding and regulating *cytR*. Collectively, our findings enhance our understanding of how ArcA and CytR collaboratively modulate *V. cholerae* motility.

## Results

### ArcA repressed *V. cholerae* motility

**Fig. 1:**
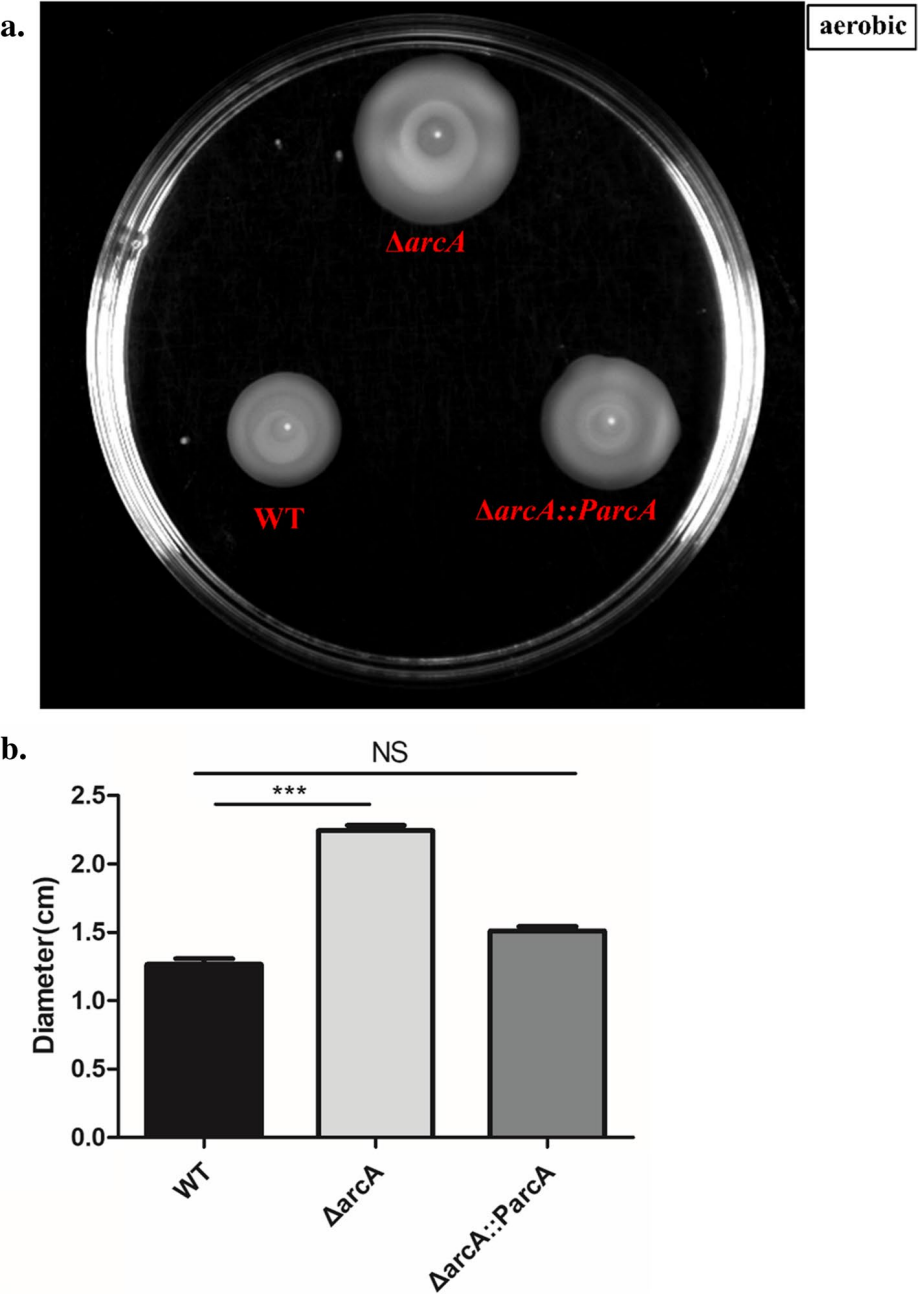
ArcA represses *V. cholerae* motility. **a.** Plate showing the motility zones of the WT strain, Δ*arcA* mutant strain, and the complementary strain Δ*arcA::ParcA* in aerobic condition. **b.** The diameter of motility zones (mean ± SEM) for nine independent biological replicates

In *E. coli* and *S. enterica sv.* Typhimurium, ArcA acts as the positive regulator of motility [13, 14]. To evaluate the role of ArcA in *V. cholerae* motility, we investigated the surface motility of Δ*arcA* mutant strain on soft agar plates in aerobic conditions. The motility zones of the Δ*arcA* mutant strain (diameter, 2.25 ± 0.15 cm) were larger than those of the WT strain (diameter, 1.27 ± 0.17 cm) (Figs. 1a and 1b). Moreover, we constructed the *arcA* complemented strain Δ*arcA::ParcA* containing a functional copy of the *arcA* sequence, using the plasmid pBAD33; and found the complementation strain (diameter, 1.51 ± 0.11 cm) restore the motility to the WT level (*p* = 0.0913) (Figs. 1a and 1b). These data indicated that ArcA functions as a negative regulator of *V. cholerae* motility.

### ArcA directly repressed *flrA* expression

**Fig. 2:**
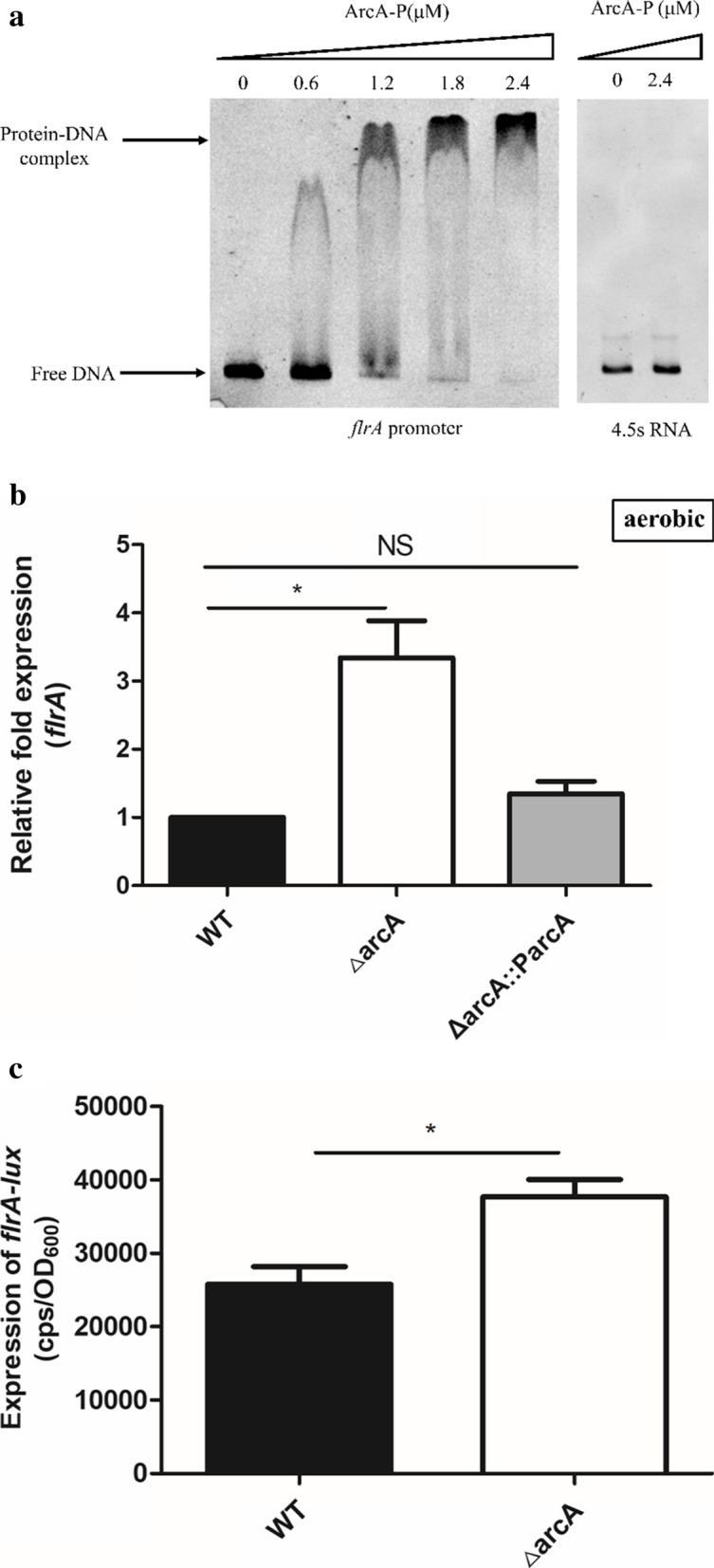
ArcA directly represses *flrA* expression. **a.** The EMSA between phosphorylated ArcA protein and the *flrA* promoter. The concentration of phosphorylated ArcA protein increased gradually (0 to 2.4 μM), and the amount of promoter DNA used in each reaction was 50 ng. 4.5S RNA served as the negative control. **b.** mRNA levels of *flrA* in the WT,,Δ*arcA* mutant strain and *arcA* complementary strain Δ*arcA::ParcA* in aerobic conditions. *, *p* < 0.05; **, *p* < 0.01; ***, *p* < 0.001; NS, no significance, *p* > 0.05. **c.** Expression of *flrA-lux* in WT strain and Δ*arcA* mutant strain. CPS (counts per second) values represent relative promoter-*lux* activities. All experiments were independently repeated at least three times. Values represent means ± SEM

The increase in the motility of the Δ*arcA* mutant strain could be attributed to lack of flagella (fla phenotype), loss of motility (mot phenotype), or loss of chemotaxis (che phenotype) [14, 23]. As previous reports, ArcA is proposed to bind to a conservative sequence (5’-TGTTA-3’) in *E. coli* [24]. Then, A search for the conservative ArcA binding sites in *E. coli* at *flrA* promoter region was performed using Virtual Footprint 3.0. We found a similar sequence (TGTTC-AAACGGTGCAACCACAACT-TCTTA) with a 19 base-spacing at positions -14 to -42 on the upstream region of *flrA* that ArcA probably binds (Fig. S10). To determine the precise cause, the EMSA screening was performed to assess whether ArcA directly binds to the promoter region of the 16 different core regulons in the flagellar heirarchy, and found that the phosphorylated ArcA directly bound to the promoter region of *flrA* (Fig. 2a), not with the other 15 ones. The following qRT-PCR indicated that *flrA* expression was increased by approximately 3.4-fold in the Δ*arcA* mutant strain compared with that in the WT strain in aerobic conditions (Fig. 2b). Moreover, we constructed the *arcA* complemented strain Δ*arcA::ParcA* containing a functional copy of the *arcA* gene, using the plasmid pBAD33; and found *flrA* expression in the complementation strain was restored to the WT level (*p* = 0.1320) (Fig. 2b). Furthermore, the promoter-lux fusion reporter plasmid was constructed with the promoter of *flrA* inserted into pMS402, and found that the activity of *flrA* promoter-lux was approximately up-regulation by 1.5-fold in the Δ*arcA* mutant strain compared to that in the WT (Fig. 2c). These data indicated that ArcA functions as a negative regulator of *V. cholerae* motility by directly repressing the expression of *flrA*.

### CytR enhanced the *fliK* expression and activated by CRP

Previous studies reported that the Δ*cytR* mutant strain shows reduced motility and the expression of flagellar-synthesis regulatory genes *flrBC* and class III flagellar-synthesis genes were reduced in the Δ*cytR* mutant strain [22]. We hypothesized that CytR modulates flagellar regulatory genes by directly binding to their promoter regions. CytR is proposed to bind to octameric inverted or direct repeats containing the consensus half sites (5’-TGCAA-3’) with variable spacing in *E. coli* [16]. We found a similar sequence (TGCAA-TAAAACCTTCACTTGGCTTACTTC-TTGCT) with a 24 base-spacing at positions -15 to -48 on the upstream region of *fliK* that CytR probably binds (Fig S11). This site overlays 18 bases of CRP-binding site (TGGAT-GCAATAAAACCT-TCACT) located at position -31 to -52 (Fig. S2) [24].

**Fig. 3:**
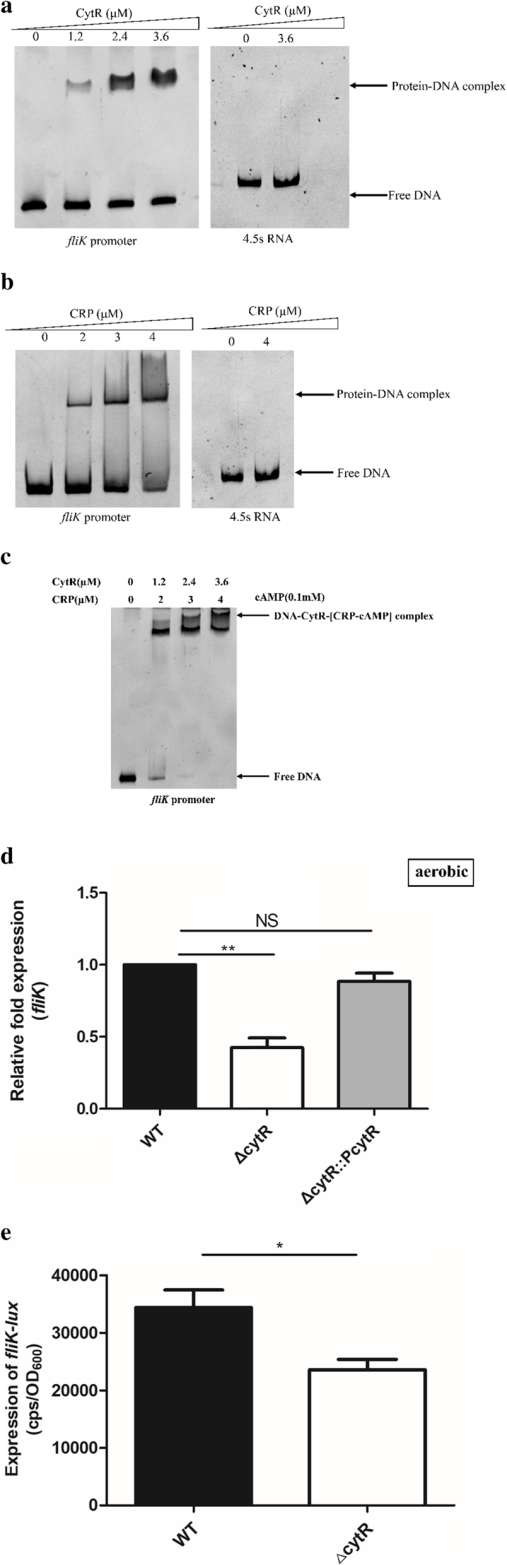
CytR directly promotes *fliK* expression. **a.** The EMSA of CytR with the *fliK* promoter. The concentration of CytR protein increased gradually (0 to 3.6 μM), and the amount of promoter DNA used in each reaction was 50 ng. **b.** The EMSA of CRP with the *fliK* promoter. The concentration of CRP protein increased gradually (0 to 4 μM), and the amount of promoter DNA used in each reaction was 50 ng. The concentration of cAMP used in each reaction was 0.1 mM. **c.** The EMSA of CytR and CRP with the *fliK* promoter. The concentration of CytR and CRP protein increased gradually (0 to 3.6 or 4 μM), and the amount of promoter DNA used in each reaction was 50 ng. The concentration of cAMP used in each reaction was 0.1 mM. **d.** mRNA levels of *fliK* in the WT, Δ*cytR* mutant strain and the complementary strain Δ*cytR::PcytR*. *, *p* < 0.05; **, *p* < 0.01; ***, *p* < 0.001; NS, no significance, *p* > 0.05. **e.** Expression of *fliK-lux* in WT strain and Δ*cytR* mutant strain. CPS (counts per second) values represent relative promoter-*lux* activities. All experiments were independently repeated at least three times. Values represent means ± SEM

The EMSA was performed to assess whether CytR directly binds to the promoter region of the 16 different core regulons in the flagellar heirarchy, and found that the CytR protein directly bound to the promoter region of *fliK* (Fig. 3a), not with the other 15 ones. Studies of gene regulation have revealed that the CRP protein and the CytR regulator can act either to repress or to activate transcription depending on the context [18]. When CytR binds to the promoter region of target genes, it forms a complex with CRP and RNA polymerase [25]. To determine if the transcriptional regulation of *fliK* by CytR occurs in the same manner as that of the above cases, we also observed CRP protein only (Fig. 3b) and co-binding of the CytR and CRP proteins by EMSA assay (Fig. 3c).

qRT-PCR revealed that *fliK* expression was approximately down-regulated by 2.4-fold in the Δ*cytR* mutant strain compared with that in the WT strain in aerobic conditions (Fig. 3d). Moreover, we constructed the *cytR* complemented strain Δ*cytR::PcytR* containing a functional copy of the *cytR* gene, using the plasmid pBAD33; and found *fliK* expression in the complementation strain was restored to the WT level (*p* = 0.1126) (Fig. 3d). Furthermore, the promoter-lux fusion reporter plasmid was constructed with the promoter of *fliK* inserted into pMS402 and found that the activity of *fliK* promoter-lux was approximately down-regulation by 1.5-fold in the Δ*cytR* mutant strain compared to that in the WT (Fig. 3e). As a whole, CytR and CRP cooperatively bind to the upstream region of *fliK*, then CytR enhance *fliK* expression in the presence of CRP as a modulator.

### ArcA directly repressed *cytR* expression

**Fig. 4:**
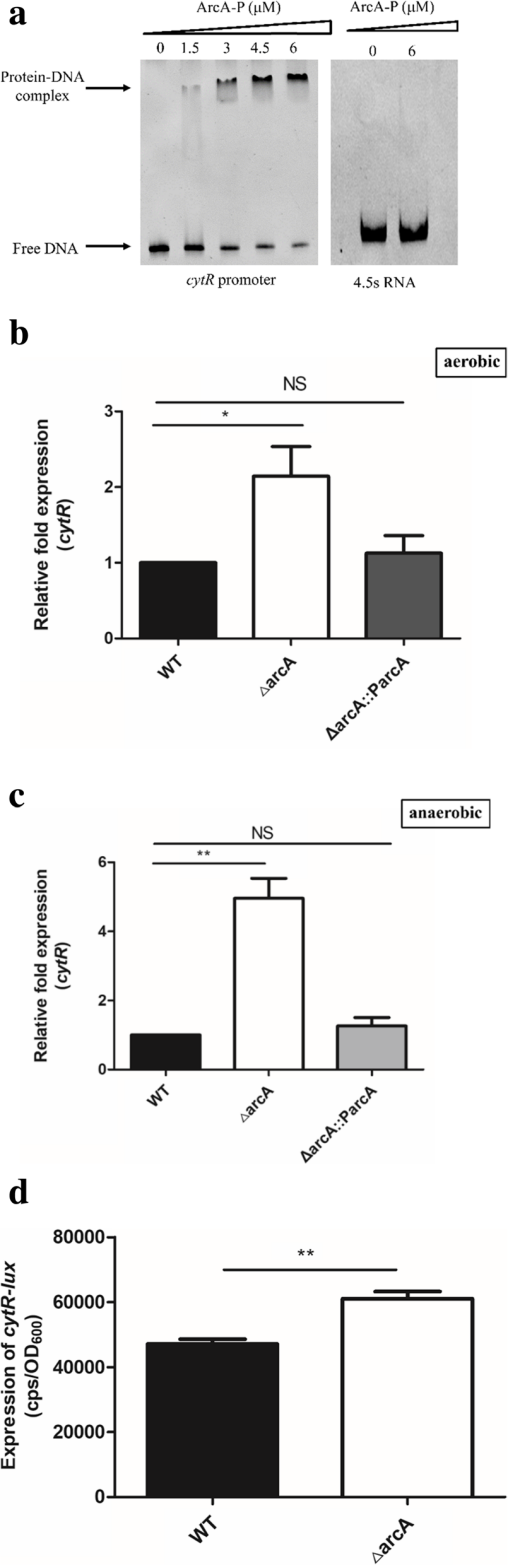
ArcA directly represses *cytR* expression. **a.** The EMSA between phosphorylated ArcA protein and the *cytR* promoter. The concentration of phosphorylated ArcA protein increased gradually (0 to 6 μM), and the amount of promoter DNA used in each reaction was 50 ng. **b.** mRNA levels of *cytR* in the WT, *ΔarcA* mutant strain and the complementary strain Δ*arcA::ParcA* in aerobic condition. *, *p* < 0.05; **, *p* < 0.01; ***, *p* < 0.001; NS, no significance, *p* > 0.05. **c.** mRNA levels of *cytR* in the WT, *ΔarcA* mutant strain and the complementary strain Δ*arcA::ParcA* in anaerobic condition. *, *p* < 0.05; **, *p* < 0.01; ***, *p* < 0.001; NS, no significance, *p* > 0.05. **d.** Expression of *cytR-lux* in WT strain and Δ*arcA* mutant strain. CPS (counts per second) values represent relative promoter-*lux* activities. All experiments were independently repeated at least three times, and the data shown represent comparable results. Values represent means ± SEM

In addition to ArcA and CytR being related to the *V. cholerae* motility, we also found that CytR is a new downstream regulatory gene of ArcA. As previous reports, ArcA is proposed to bind to a conservative sequence (5’-TGTTA-3’) in *E. coli* [24]. Then, A search for the conservative ArcA binding sites in *E. coli* at *cytR* promoter region was performed using Virtual Footprint 3.0. We found a similar sequence (TGTTA-ATTTTGTCAGCAAATTAATGC-TTATTA) with a 21 base-spacing at positions -11 to -42 on the upstream region of *cytR* that ArcA probably binds (Fig. S12). Then EMSA showed that the phosphorylated ArcA directly binds to the promoter of *cytR* (Fig. 4a). qRT-PCR was was performed in both aerobic and anaerobic conditions. The results showed that the *cytR* expression was increased by 2.1-fold in aerobic conditions, and 5.0-fold in anaerobic condition in the Δ*arcA* mutant strain compared to that in the WT strain (Fig. 4b and 4c). Moreover, we constructed the *arcA* complemented strain Δ*arcA::ParcA* containing a functional copy of the *arcA* sequence, using the plasmid pBAD33; and found *cytR* expression in the complementation strain restore to the WT level in aerobic conditions (*p* = 0.6134) and anaerobic conditions(*p* = 0.3347) (Fig. 4b and 4c). Furthermore, the promoter-lux fusion reporter plasmid was constructed with the promoter of *cytR* inserted into pMS402 and found that the activity of *cytR* promoter-lux was approximately up-regulation by 1.3-fold in the Δ*arcA* mutant strain compared to that in the WT (Fig. 4d). These data indicated that ArcA repressed *cytR* expression by directly binding to its promoter region.

## Discussion

ArcA and CytR involved in multiple regulation in *V. cholerae*. ArcA, being a regulator, its function in *V. cholerae* motility is still not known. Here, we for the first time aimed to understand the role of this multifunctional transcription factor in *V. cholerae* motility. Our results aslo suggest an expanded role of ArcA in *V. cholerae* in the manner of *cytR* and further evidence the relationship between CytR and *V. cholerae* motility.

More than 50 genes are involved in flagella synthesis and regulation in *V. cholerae* [7]. The flagella-synthesis genes in *V. cholerae* are categorized into a four transcriptional hierarchy [26]. The σ54-dependent transcriptional activator FlrA is the only class I gene in this hierarchy [27]. FlrA is the master regulator of the *V. cholerae* flagellar transcription hierarchy because it is important for the expression of all other flagellar genes. The *fliK* operon is transcribed from a class III promoter [28]. Flagellar assembly occurs in a number of stages, and in this process, the ‘‘checkpoint control’’ protein FliK functions in detecting when the flagellar hook substructure has reached its optimal length. FliK then terminates hook export and assembly and transmits a signal to begin filament export [8, 29].

**Fig. 5:**
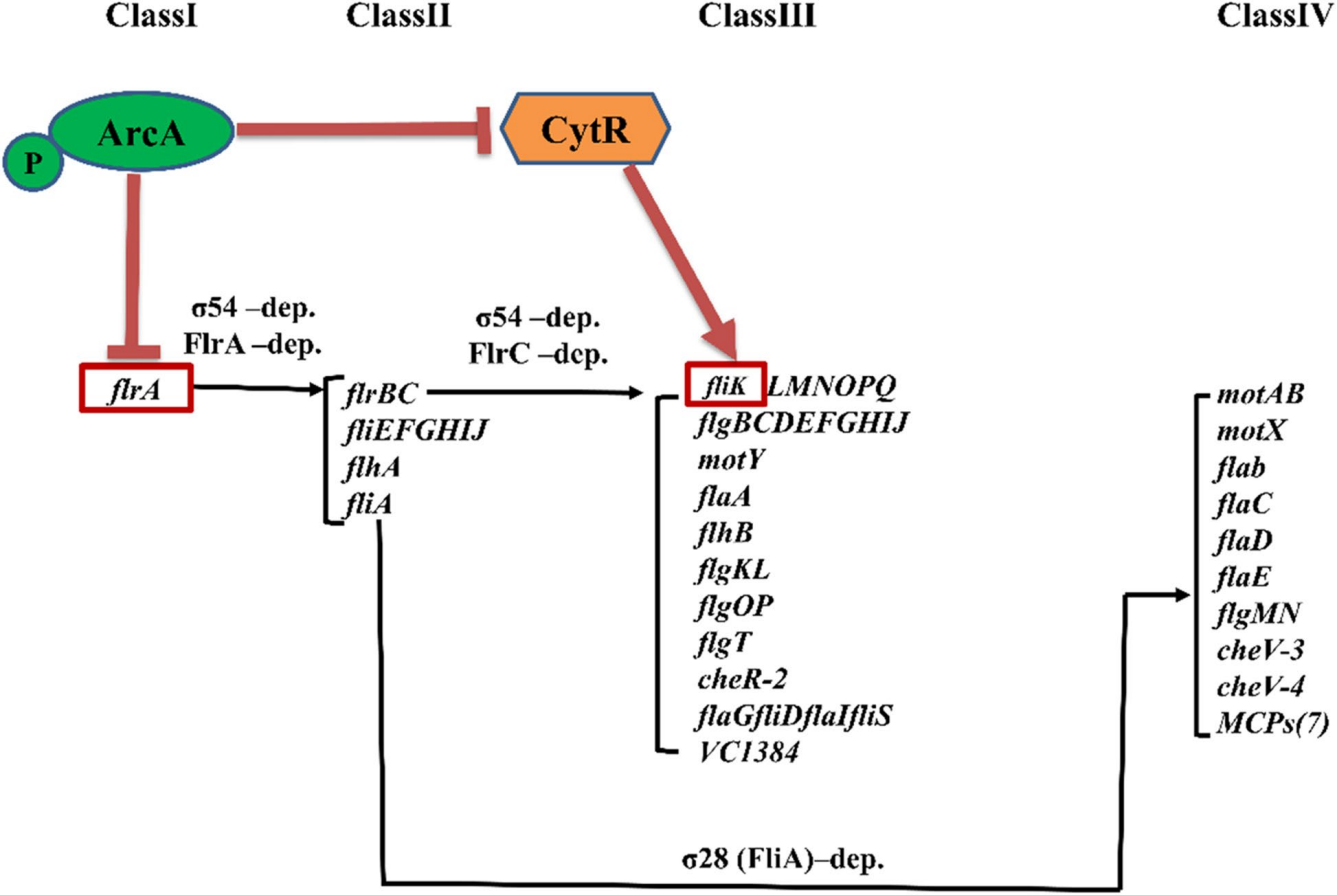
Schematic representation of *V. cholerae* motility regulation network by ArcA regulating the expression of *flrA* directly and *fliK* indirectly in the manner of *cytR*

In *E. coli*, the Δ*arcA* mutant strain has been reported to show loss of motility, with ArcA being necessary for the expression of *fliA*, but not for that of the master regulators *flhDC* [30]. In avian pathogenic *E. coli*, ArcA directly regulates the expression of *motA*, *motB*, and *cheA* [15], whereas in *S. enterica sv.* Typhimurium, it activates class II and III flagellar genes and seems to slightly repress *flhDC* [31]. In contrast, in *V. cholerae*, we found that ArcA reduced motility by a directly way of repressing the expression of the class I flagellar regulatory gene *flrA*, and an indirrectly way of repressing the expression of the class III flagellar regulatory gene *fliK* via *cytR* (Fig. 5). The expression of *cytR* was up-regulated by 2.1-fold in Δ*arcA* stain compared to the WT under the aerobic condition (Fig. 4b); likewise, up-regulated by 5.0-fold under the anaerobic condition (Fig. 4c). So the further qRT-PCR was performed on the *cytR* in Δ*arcA* stain under both the aerobic and anaerobic conditions, and the data showed that the expression of *cytR* between these two conditions was not significantly different. In other word, the repression of ArcA on *cytR* was consistent, no matter under the aerobic or anaerobic conditions.

In uropathogenic *E. coli*, CytR evidently represses motility and flagellar expression by directly binding to the upstream region of *flhD*, which encodes the master regulator for flagellar expression [20]. In the Δ*cytR* mutant strain of *V. cholerae*, qRT-PCR showed that the expression of the class II flagellar genes *flrB* and *flrC*, and that of several class III flagellar genes was downregulated [32]. We further investigated the target binding site of CytR and found that CytR bound to the promoter region of *fliK* and acts as a positive regulator of *fliK* (Fig. 5).

Our results suggest an extended role of ArcA in *V. cholerae* motility, and found its new downstream regulatory gene, *cytR*, which is also a global regulator and influences the motility by directly increasing *flik* expression.

## Conclusions

In this work, we report that the global regulators ArcA and CytR collaboratively modulate *V. cholerae* motility. Here, we provide evidence that ArcA plays a fundamental role in *V. cholerae* motility by regulating the expression of *flrA* directly and *fliK* indirectly in the manner of *cytR*.

## Materials and methods

### Bacterial strains, plasmids, and growth conditions

All strains and plasmids used in this study are shown in Table 1. In aerobic condition, all strains were grown overnight at 37 °C in lysogeny broth/agar. In anaerobic condition, bacterial cultures in an anaerobic incubator were grown in the presence of 1 g/l cysteine and 1 mg/l resazurin. Antibiotics were added, as required 40 μg/ml, polymyxin B or 25 μg/ml, chloramphenicol. All chemicals were purchased from Sigma (St. Louis, MO, USA).

**Table 1:** Bacterial strains and plasmids used in this study

Strain or plasmid	Characteristics^a^	Reference or source
Vibrio cholerae		
El2382	Virulent strain, O1 El Tor type, PmB^r^	Shanghai Municipal Center for Disease Control & Prevention
Δ*arcA*	El2382, deletion of *arcA*, PmB^r^	This study
Δ*arcA::ParcA*	Δ*arcA* containing pBAD33 carrying *arcA* ORF with its own promoter, PmB^r^	This study
Δ*cytR*	El2382, deletion of *cytR*, PmB^r^	This study
Δ*cytR::PcytR*	Δ*cytR* containing pBAD33 carrying *cytR* ORF with its own promoter, PmB^r^	This study
Escherichia coli		
S17-1 (λpir)	*Tpr Smr recA thi pro rK- mK- RP4:2-Tc:MuKm Tn7 λ pir* (*thi pro hsdR hsdM* + *recA RP4-2-Tc:: Mu-Km-Tn7*)	[1]
BL21(DE3)	Host strain for protein expression	This study
BL21/pET28a::*arcA*	BL21(DE3) with pET28a carrying the *arcA* ORF, Km^r^	This study
BL21/pET28a::*cytR*	BL21(DE3) with pET28a carrying the *cytR* ORF, Km^r^	This study
BL21/pET28a::*crp*	BL21(DE3) with pET28a carrying the *crp* ORF, Km^r^	This study
Plasmids		
pRE112	pGP704 suicide plasmid, pir dependent, *oriT*, *oriV*, *sacB*, Cm^r^	[2]
pBAD33	arabinose inducible promoter, Cm^r^	[3]
pET28a	Expression vector, Km^r^	This study
pET28a::*arcA*	pET28a carrying the *arcA* ORF, Km^r^	This study
pET28a::*cytR*	pET28a carrying the *cytR* ORF, Km^r^	This study
pET28a::*crp*	pET28a carrying the *crp* ORF, Km^r^	This study
pMS402	For construct promoter-*lux*CDABE reporter fusion; Km^r^	This study
*cytR-lux*	pMS402 carrying the *cytR* promoter region, Km^r^	This study
*flrA-lux*	pMS402 carrying the *flrA* promoter region, Km^r^	This study
*fliK-lux*	pMS402 carrying the *fliK* promoter region, Km^r^	This study

^a^ r resistant. Cm, chloramphenicol, PmB, polymyxinB, Km, kanamycin

### Construction of the deletion mutant of ArcA and its complementation

The ArcA isogenic deletion mutant was constructed using the suicide plasmid pRE112 method, as previously described [33]. Briefly, 1) the recombinant plasmid pRE112-Δ*arcA*-*V.cholerae* was constructed and transformed into *E. coli* λpir; 2) intergeneric conjugation between *E. coli* and *V. cholerae*. DNA sequencing was performed to confirm the sequences of the constructed deletion plasmids. The Δ*arcA* mutant strain were complemented with the plasmid cloned into the vector pBAD33. The complemented strain was constructed using a previously reported procedure [34]. Table 2 lists the primers used in this study.

**Table 2:** Primers used in this study

Name	Sequence (5′–3′)	Amplifified fragment
Primers for construction of mutants		
∆*arcA*-S-F	GCTCTAGACGATCAAGCATTGCTGTAAA	∆*arcA*-S (500)
∆*arcA*-S-R	AAAGAAGAGGTAGCGTTACCTAAACTTGTGA	
∆*arcA*-X-F	GGTAACGCTACCTCTTCTTTTATATCTAATTAG	∆*arcA*-X (500)
∆*arcA*-X-R	CGGAGCTCAACATCATGCCGGTGAGAG	
		∆*arcA*-SX (1000)
*arcA*-F	ATGCAAACCCCGCAGATCCTT	*arcA* (717)
*arcA*-R	TTAATCTTCTAAATCACCACAG	
∆*cytR*-S-F	CGGGGTACCTCCGAGGACGACACGATAC	∆ *cytR*-S (512)
∆ *cytR*-S-R	GTAAAAATACCCCACCTTCGAAACCGA	
∆ *cytR*-X-F	CGAAGGTGGGGTATTTTTACCCTCTTTTCTCTATCG	∆ *cytR*-X (534)
∆ *cytR*-X-R	CGAGCTCTTTCGAGCTGAAGCCAATC	
		∆ *cytR*-SX (1046)
*cytR*-F	ATGGCGACAATGAAGGATGT	*cytR* (1015)
*cytR*-R	AGGTGGGTTACTTCTTGCTTG	
Primers for identification of plasmid		
pRE112-U-F	CACTGTTCGTCCATTTCCG	pRE112-UD (567)
pRE112-D-R	TTCGTCTCAGCCAATCCCT	
		pRE112-U-*arcA*-D (1284)
		pRE112-U-*cytR*-D (1582)
pBAD33-U-F	AACAAAGCGGGACCAAAG	pBAD33-UD (529)
pBAD33-D-R	AGAGCGTTCACCGACAAA7	
		pBAD33-U-*arcA*-D (1246)
		pBAD33-U-*cytR*-D (1544)
pET28a-U-F	TAATACGACTCACTATAGGG	pET28a-UD (318)
pET28a-D-R	GCTAGTTATTGCTCAGCGG	
		pET28a-U-*arcA*-D (1035)
		pET28a-U-*cytR*-D (1395)
		pET28a-U-*crp*-D (918)
Primers for construction of complemented strain		
*∆arcA::ParcA*-F	CGAGCTCTAAATCAACAAAGTGATTGGAA	*∆arcA::ParcA* (732)
*∆arcA::ParcA*-R	GGGGTACCTTAATCTTCTAAATCACCACAG	
*∆cytR::PcytR*-F	GGGGTACCATGGCGACAATGAAGGATGTTG	*∆cytR::PcytR* (1031)
*∆ cytR::P cytR* -R	GCTCTAGAGCTTACTTCTTGCTTGGCGGCG	
Primers for protein cloning		
pET28a-*arcA*-F	CGGGATCCATGCAAACCCCGCAGATCCT	pET28a-*arcA* (734)
pET28a-*arcA*-R	CCGCTCGAGTTAATCTTCTAAATCACCAC	
pET28a-*cytR*-F	CGCGGATCCATGGCGACAATGAAGGATG	pET28a-*cytR* (1095)
pET28a-*cytR*-R	CCGCTCGAGTTACTTCTTGCTTGGCGG	
pET28a-*crp*-F	CGCGGATCCATGGTTCTAGGTAAACCTCA	pET28a-*crp* (617)
pET28a-*crp*-R	CCGCTCGAGTTAGCGAGTGCCGTAAACCA	
Primers for bioluminescent reporter assays		
pMS402-*cytR*-F	CGGGATCCCTTTTACTACAAACGCCGAAT	pMS402-*cytR* (1032)
pMS402-*cytR*-R	CCGCTCGAGGTATTTTTACCCTCTTTTCTCTATC	
pMS402-*flrA*-F	CGGGATCCAACGTTTAGGTAAAGCCTTGG	pMS402- *flrA* (1484)
pMS402-*flrA*-R	CCGCTCGAGAGGTGAGATTATTTGCCTTTATTAT	
pMS402-*fliK*-F	CGGGATCCACTGCGTCAAATTGAACAGTACC	pMS402-*fliK* (2042)
pMS402-*fliK*-R	CCGCTCGAGAGTGGAATTGAAGTCTGAGCATG	
Primers for qRT-PCR		
16S rRNA-F	GTGTACGGTGAAATGCGTAGAG	275 bp
16S rRNA-R	GCGTGGACTACCAGGGTATCTAAT	
qRT- *cytR* -F	ATTCGCGGTATTGAAGATGC	189 bp
qRT- *cytR* -R	AGGCGGTAGGTTTTTCTGCT	
qRT- *flrA* -F	CCTGAAGGGGTGAATCTCAA	157 bp
qRT- *flrA* -R	GCATGTTGTATTTGCGCATC	
qRT- *fliK* -F	CTCAAACCGTAGCGGTCAAT	235 bp
qRT- *fliK* -R	TGTACCAGTTGCGACTCAGC	
Primers for EMSA		
EMSA-*cytR*-F	ATCGCGTTTTATAACGCTGAT	200 bp
EMSA-*cytR*-R	CTAGAAATCATGGCCATAACCA	
EMSA- *flrA* -F	ATAAAGTCAGCTTGGGATCAAA	300 bp
EMSA- *flrA* -R	AGGTGAGATTATTTGCCTTTATTAT	
EMSA- *fliK* -F	GTCAAAAACGGAAATCCTATCA	300 bp
EMSA- *fliK* -R	AGTGGAATTGAAGTCTGAGCAT	

Underlined letters show Xba I、Sac I、Kpn I、BamH I or XhoI restriction site

F/R: upstream and downstream primers of gene,S/X-F/R: The upstream and downstream primers for the upstream and downstream gene fragments of *arcA* in the E12382 genome, U/D-F/R: Upstream and downstream sequencing primers of plasmid

### Soft agar motility assay

*V. cholerae* strains were grown in LB broth for overnight and inoculated (1 μl) into freshly poured 0.3% agar plates, followed by incubation and grown at 30 °C for 24 h. The diameters of motility zones at least six independent colonies were averaged [35, 36].

### RNA isolation and quantitative real time PCR (qRT-PCR)

Bacterial cultures were grown in LB medium aerobically or anaerobically at 37 °C to the mid-logarithmic phase (OD_600_ approximately 0.6). Total RNA was extracted using TRIzol (Invitrogen, Waltham, MA, USA, #15,596–018), as per manufacturer instructions. cDNA was synthesized using a Prime Script RT Reagent Kit with gDNA Eraser (Takara, Shiga, Japan). qRT-PCR was performed on an Applied Biosystems 7500 sequence detection system with SYBR green fluorescence dye. The 16 s rRNA gene was used as the reference control for sample normalization [37]. Table 2 lists the primers used in this study. The relative expression levels of target transcripts were calculated according to the 2^−∆∆CT^ method [38]. Each experiment was performed in triplicate. Expression changes of > twofold with *p* < 0.05 were considered statistically significant.

### Electrophoretic mobility shift assay (EMSA)

A sequence encoding a ArcA/CytR/CRP-His_6_ fusion protein was cloned into vector pET-28a, expressed in *E. coli* BL21 (DE3), and purified using an Ni–NTA-Sefinose Column in accordance with the protocol provided by the manufacturer [37, 39]. EMSA was performed by adding increasing amounts of purified phosphorylated ArcA protein (0, 1.5, 3.0, 4.5, and 6.0 µM or 0, 0.6, 1.2, 1.8 and 2.4 µM) to *cytR* or *flrA* DNA fragments (50 ng) in a binding buffer [10 mM Tris–HCl (pH 8.0), 1 mM EDTA, 1 mM DTT, 50 mM KCl, 50 μg/mL BSA, 10% glycerol] supplemented with 20 nM acetyl phosphate [39], followed by incubation for 40 min at room temperature. Similarly, *fliK* DNA fragments (50 ng) were incubated with increasing amounts of 6 × His-tagged CytR or CRP protein (0, 1.2, 2.4, and 3.6 µM or 0, 2, 3, and 4 µM) in a binding buffer [20 mM Tris–HCl (pH 7.5), 50 mM KCl, 1 mM dithiothreitol, 200 µM cAMP, and 10% glycerol] [20], followed by incubation for 40 min at room temperature. The concentration of cAMP used in each reaction was 0.1 mM. 4.5 s RNA served as the negative control. The reaction mixtures were then electrophoresed on a 6% native polyacrylamide gel. Protein was visualized using a Typhoon phosphorimager (GE Healthcare, Chicago, IL, USA).

### Bioluminescent reporter assays

The procedures of the lux bioluminescent reporter assay were described in previous study [40]. Briefly, bacterial cultures were grown in LB medium at 37 °C to the mid-logarithmic phase (OD_600_ approximately 0.6). The cultures were transferred into a black 96-well plate with a transparent bottom. Promoter activities were measured and bacterial growth was measured by OD_600_ in a Synergy 2 plate reader (BioTek) at the same time.

### Statistical analyses

All data are expressed as means ± standard deviation (SD). Differences between two groups were evaluated using independent-samples t-test or Mann–Whitney U test. Values of *p* ≤ 0.05, 0.01, or 0.001 were considered to be statistically significant (*), highly significant (**), or extremely significant (***), respectively.

## Supplementary Information

**Additional file 1.**

## Data Availability

All data generated or analyzed during this study are included in this published article.

## Abbreviations

ArcA: Anoxic redox control cognate response regulator
CRP: Cyclic-AMP receptor protein
CytR: Cytidine repressor
WT: Wild-type
ΔarcA: ArcA isogenic deletion mutant strain;
ΔarcA: ParcA, complementation strain of arcA
LB: Luria–Bertani
EMSA: Electrophoretic mobility shift
