# Supplementary material for: The global regulators ArcA and CytR collaboratively modulate Vibrio cholerae motility
Source: BMC Microbiol. 2022 Jan 12;22:22. doi: 10.1186/s12866-022-02435-y (PMC8753867; doi:10.1186/s12866-022-02435-y)
Supplement: Supplementary file 1 — Additional file 1. [file 12866_2022_2435_MOESM1_ESM.docx]

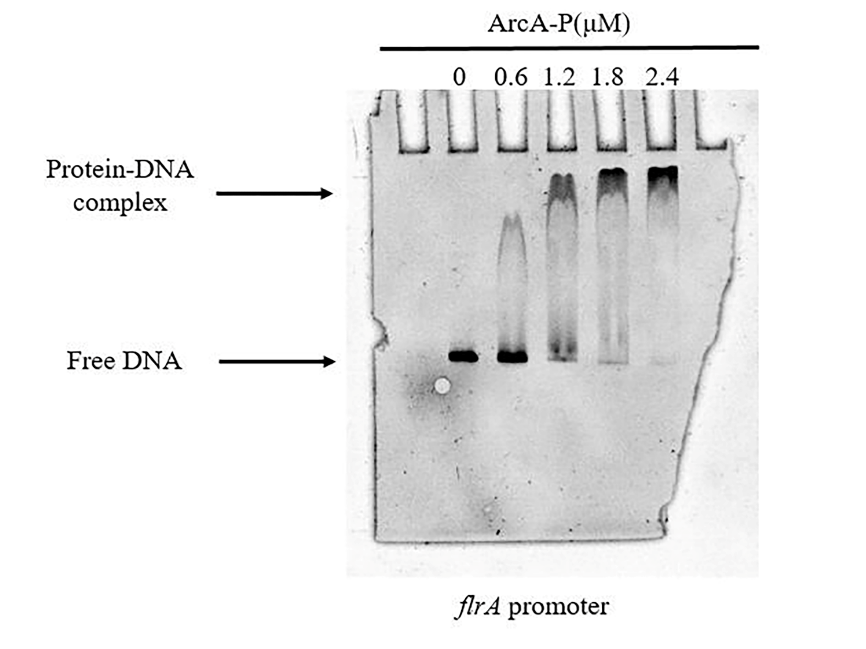


**Fig. S1** The EMSA between phosphorylated ArcA protein and the *flrA* promoter.The concentration of phosphorylated ArcA protein (ArcA-P) increased gradually (0 to 2.4 μM), and the amount of promoter DNA used in each reaction was 50 ng. Fig. 2a (left) in manuscript was cropped from Fig. S1.


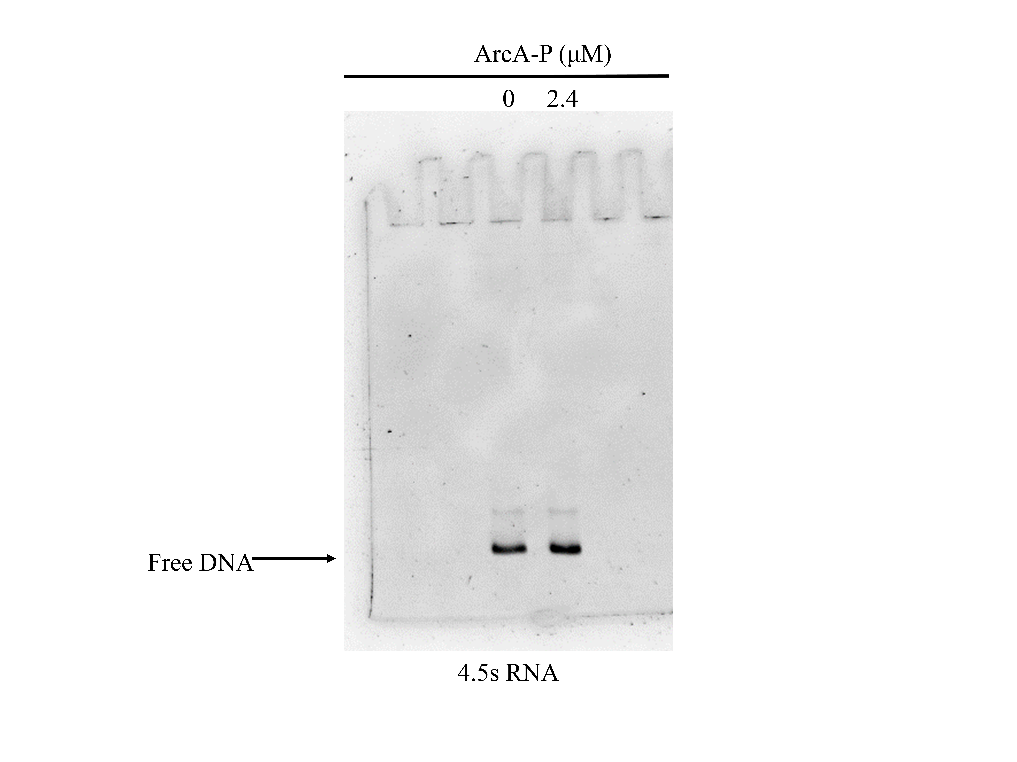


**Fig. S2** The EMSA between phosphorylated ArcA protein and the 4.5s RNA.The concentration of phosphorylated ArcA protein (ArcA-P) increased gradually (0 to 2.4 μM), and the amount of promoter DNA used in each reaction was 50 ng. Fig. 2a (right) in manuscript was cropped from Fig. S2.


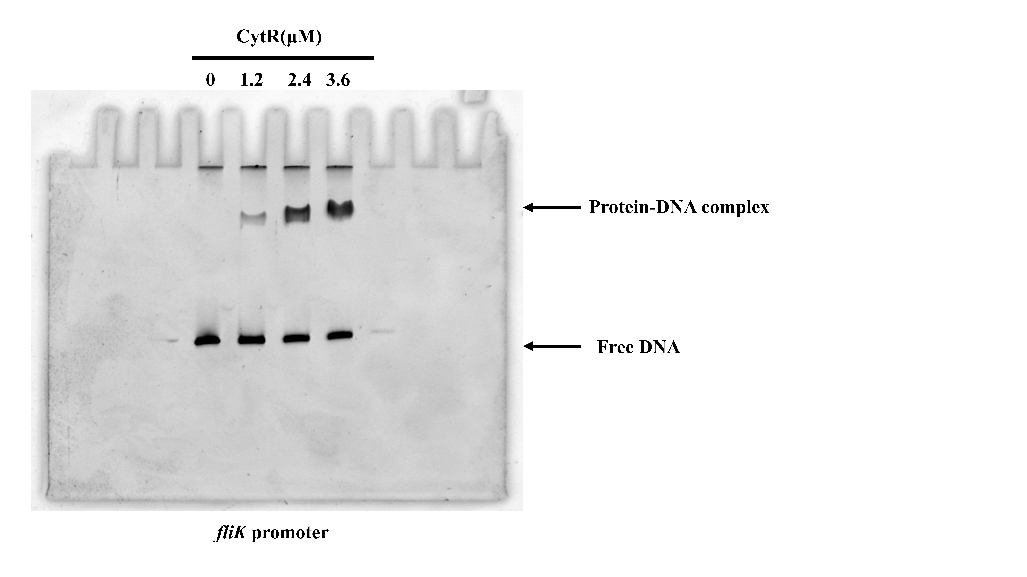


**Fig. S3** The EMSA between CytR protein and the *fliK* promoter.The concentration of phosphorylated CytR protein increased gradually (0 to 3.6 μM), and the amount of promoter DNA used in each reaction was 50 ng. Fig. 3a (left) in manuscript was cropped from Fig. S3.


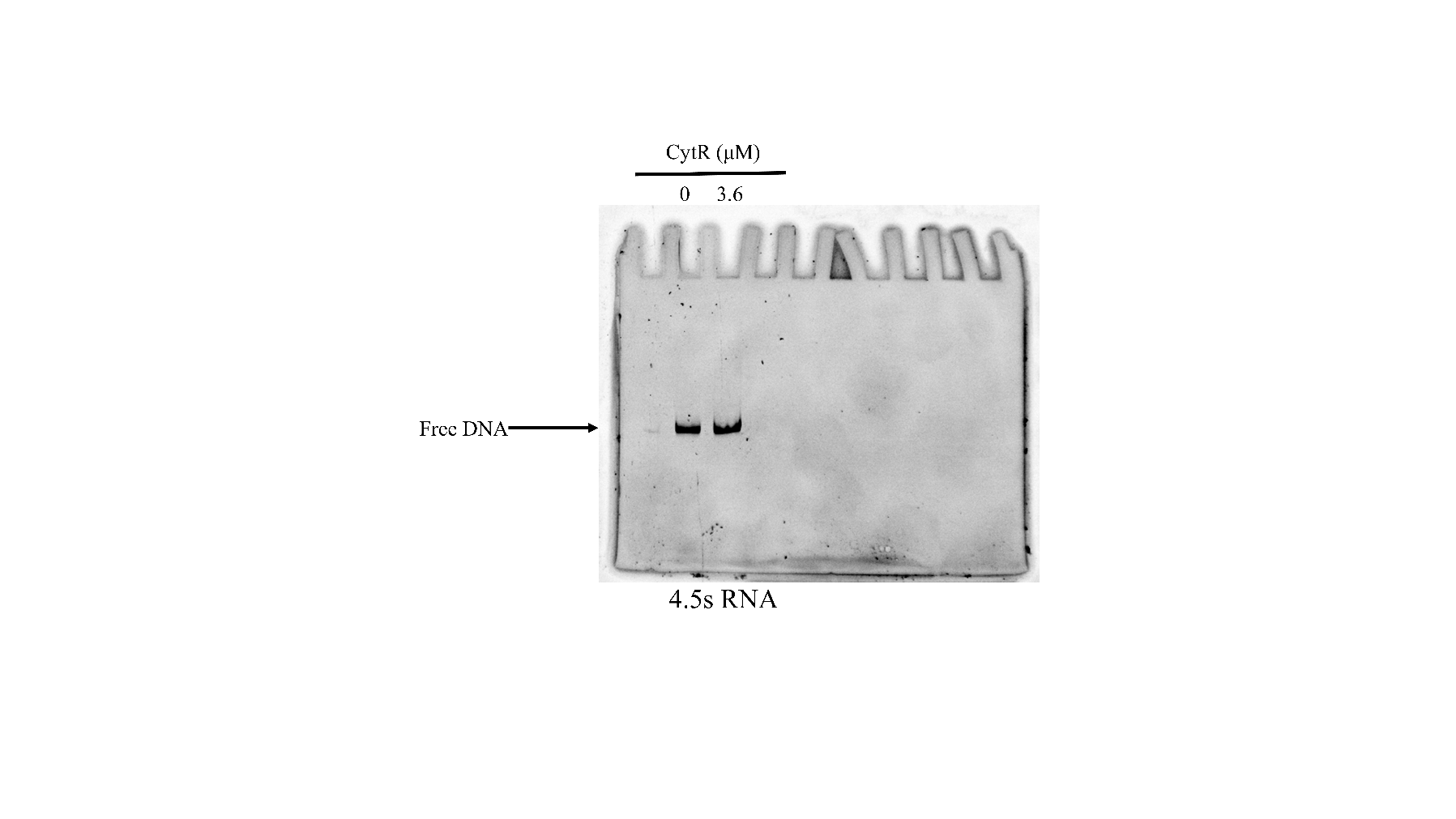
**Fig. S4** The EMSA between CytR protein and the 4.5sRNA.The concentration of CytR protein increased gradually (0 to 3.6 μM), and the amount of promoter DNA used in each reaction was 50 ng. Fig. 3a (right) in manuscript was cropped from Fig. S4.


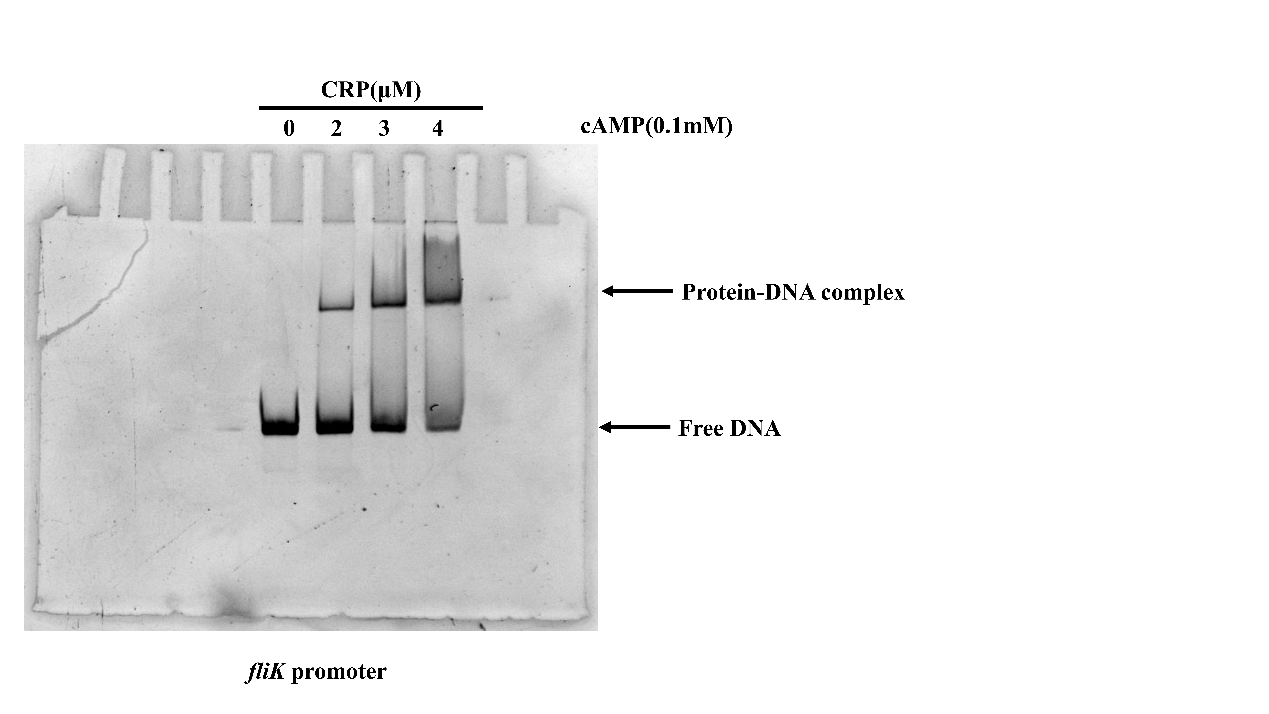


**Fig. S5** The EMSA between CRP protein and the *fliK* promoter.The concentration of phosphorylated CRP protein increased gradually (0 to 4 μM), and the amount of promoter DNA used in each reaction was 50 ng. The concentration of cAMP used in each reaction was 0.1mM. Fig. 3b (left) in manuscript was cropped from Fig. S5.

**Fig. S6** The EMSA between CRP protein and the 4.5s RNA.The concentration of CRP protein increased gradually (0 to 4 μM), and the amount of promoter DNA used in each reaction was 50 ng. Fig. 3b (right) in manuscript was cropped from Fig. S
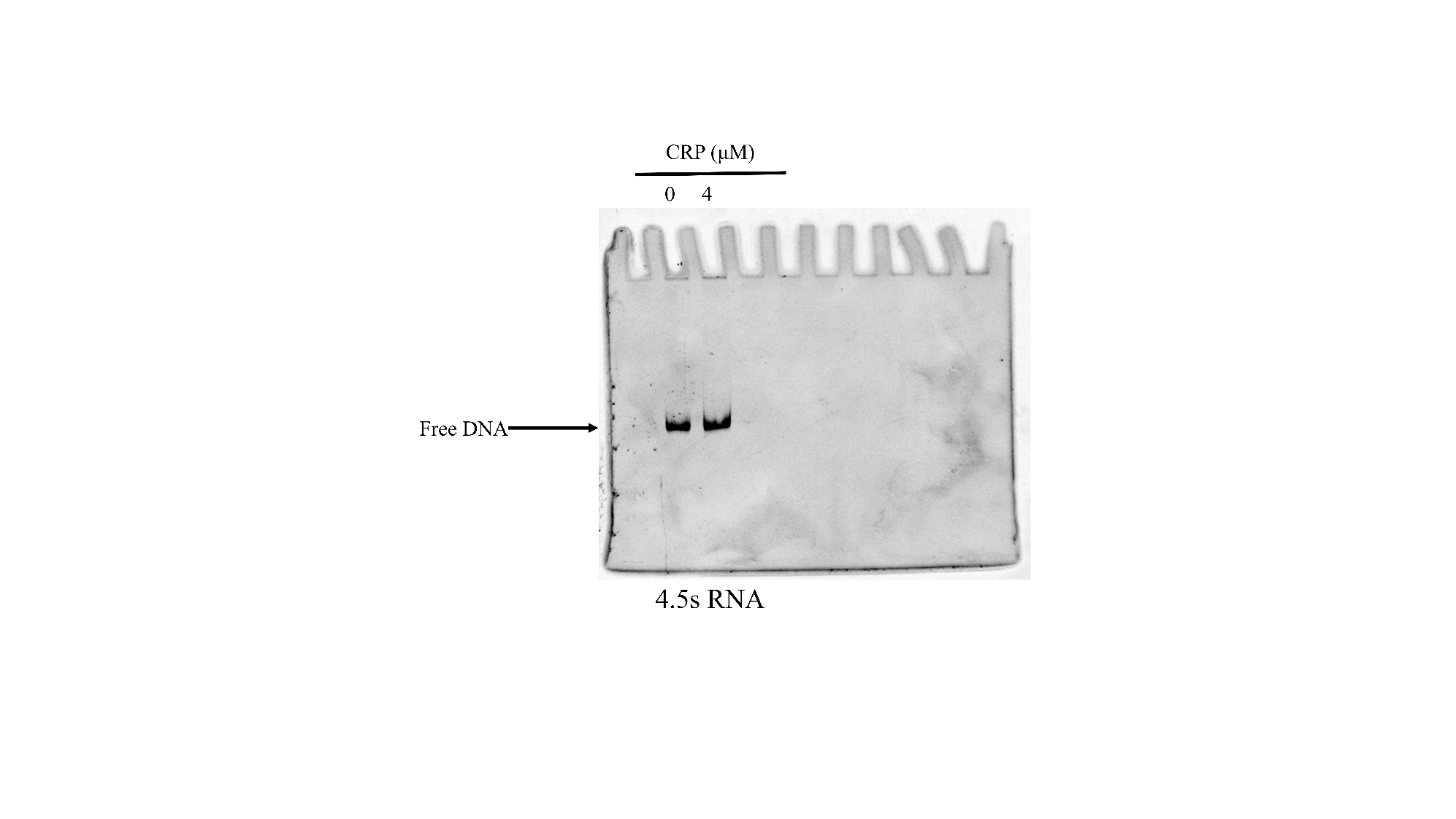
6.


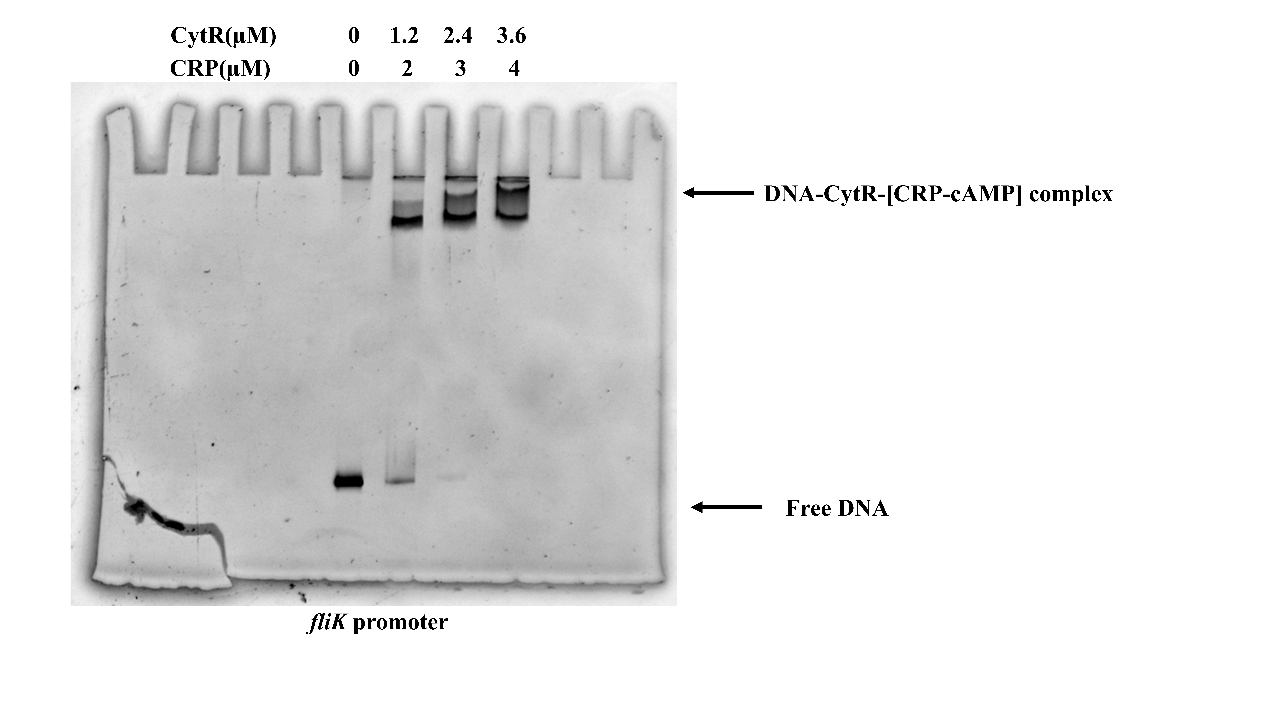


**Fig. S7.** The EMSA of both CytR and CRP with the *fliK* promoter. The concentration of CytR and CRP protein increased gradually (0 to 3.6 or 4 μM), and the amount of promoter DNA used in each reaction was 50 ng. The concentration of cAMP used in each reaction was 0.1 mM. Fig. 3c in manuscript was cropped from Fig. S7.


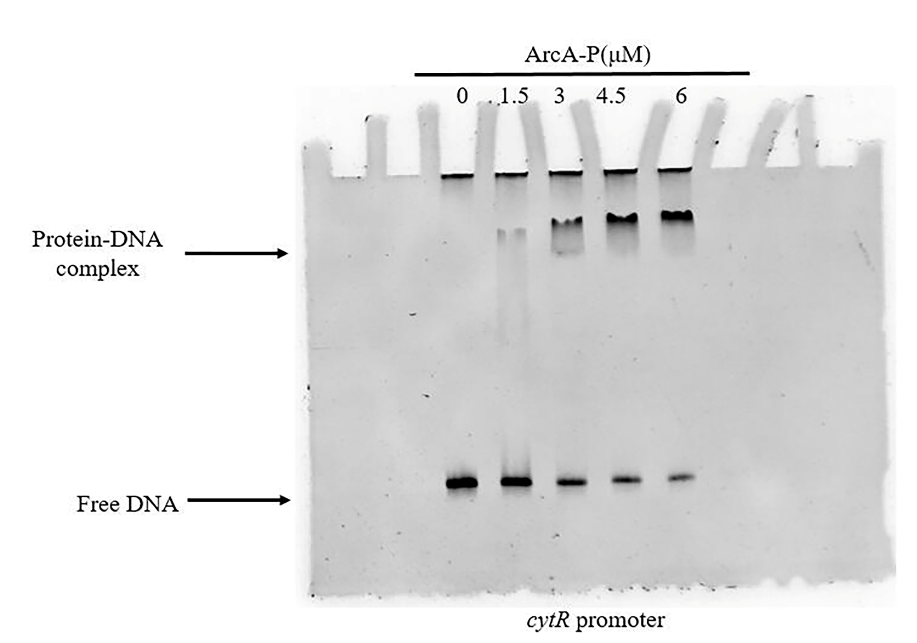


**Fig. S8** The EMSA between phosphorylated ArcA protein and the *cytR* promoter.The concentration of phosphorylated ArcA protein (ArcA-P) increased gradually (0 to 6 μM), and the amount of promoter DNA used in each reaction was 50 ng. Fig. 4a (left) in manuscript was cropped from Fig. S8.


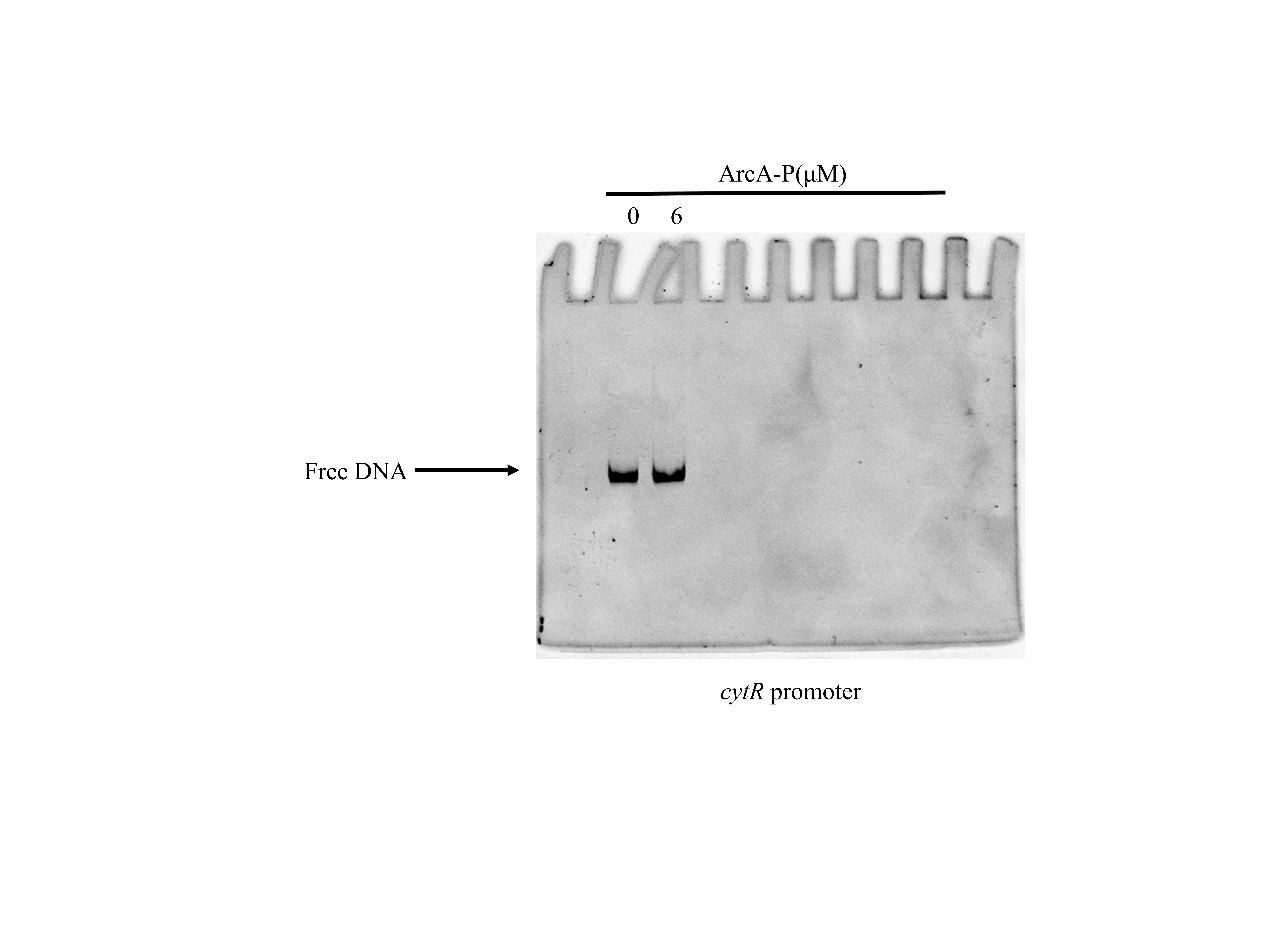


4.5s RNA

.

**Fig. S9** The EMSA between phosphorylated ArcA protein and 4.5s RNA.The concentration of phosphorylated ArcA protein increased gradually (0 to 6 μM), and the amount of promoter DNA used in each reaction was 50 ng. Fig. 4a (right) in manuscript was cropped from Fig. S9.


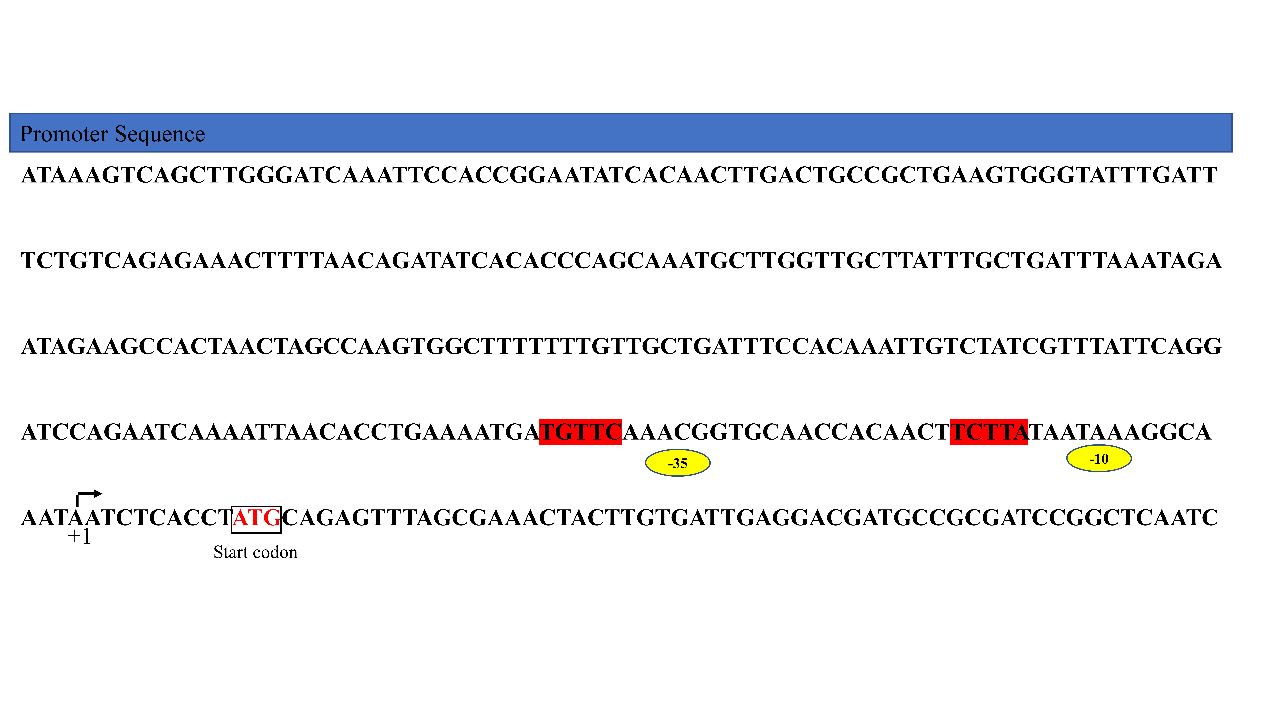


**Fig. S10** Diagram showing the promoter region of the *flrA* gene. The two putative ArcA binding sites at the *flrA* promoter region are in red. The transcription start site is labeled as +1.


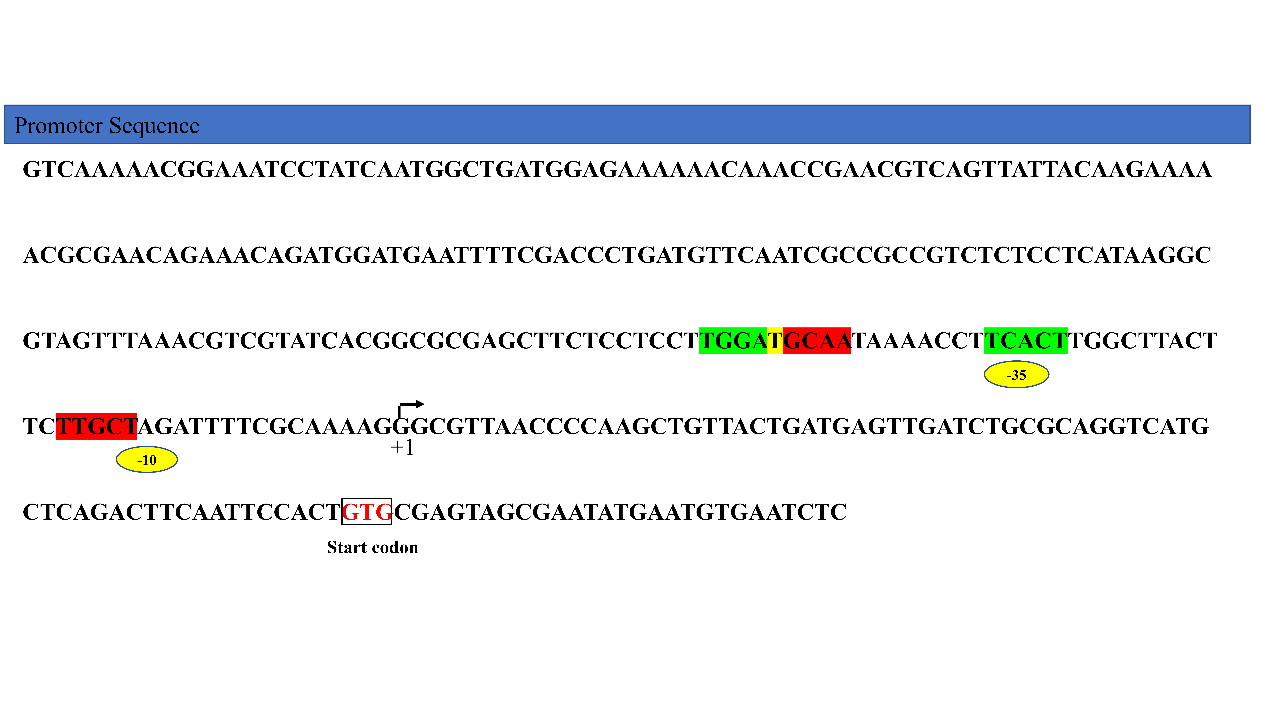


**Fig. S11** Diagram showing the promoter region of the *fliK* gene. The putative two CytR and two CRP binding sites at the *fliK* promoter region are in red and green, respectively. The transcription start site is labeled as +1.


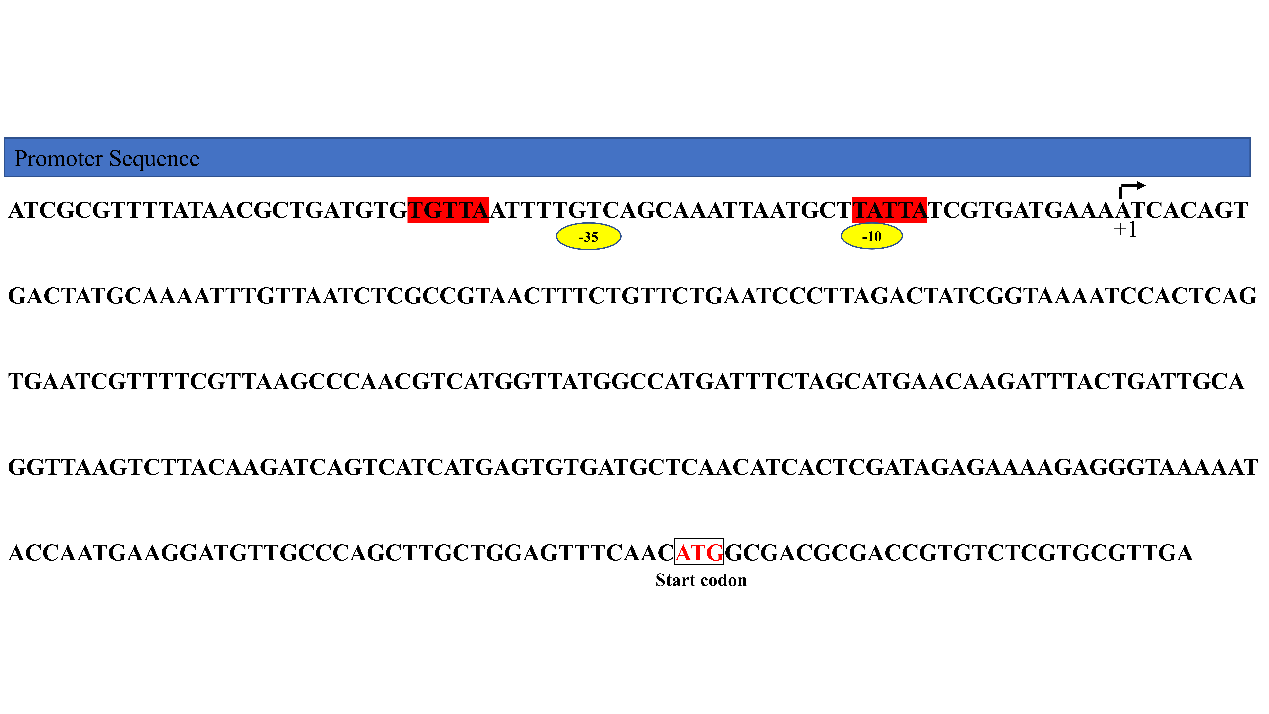


**Fig. S12** Diagram showing the promoter region of the *cytR* gene. The two putative ArcA binding sites at the *cytR* promoter region are in red. The transcription start site is labeled as +1.
